# Supplementary figures and images for: Transcriptomic characterization revealed that METTL7A inhibits melanoma progression via the p53 signaling pathway and immunomodulatory pathway
Source: PeerJ. 2023 Aug 2;11:e15799. doi: 10.7717/peerj.15799 (PMC10404031; doi:10.7717/peerj.15799)

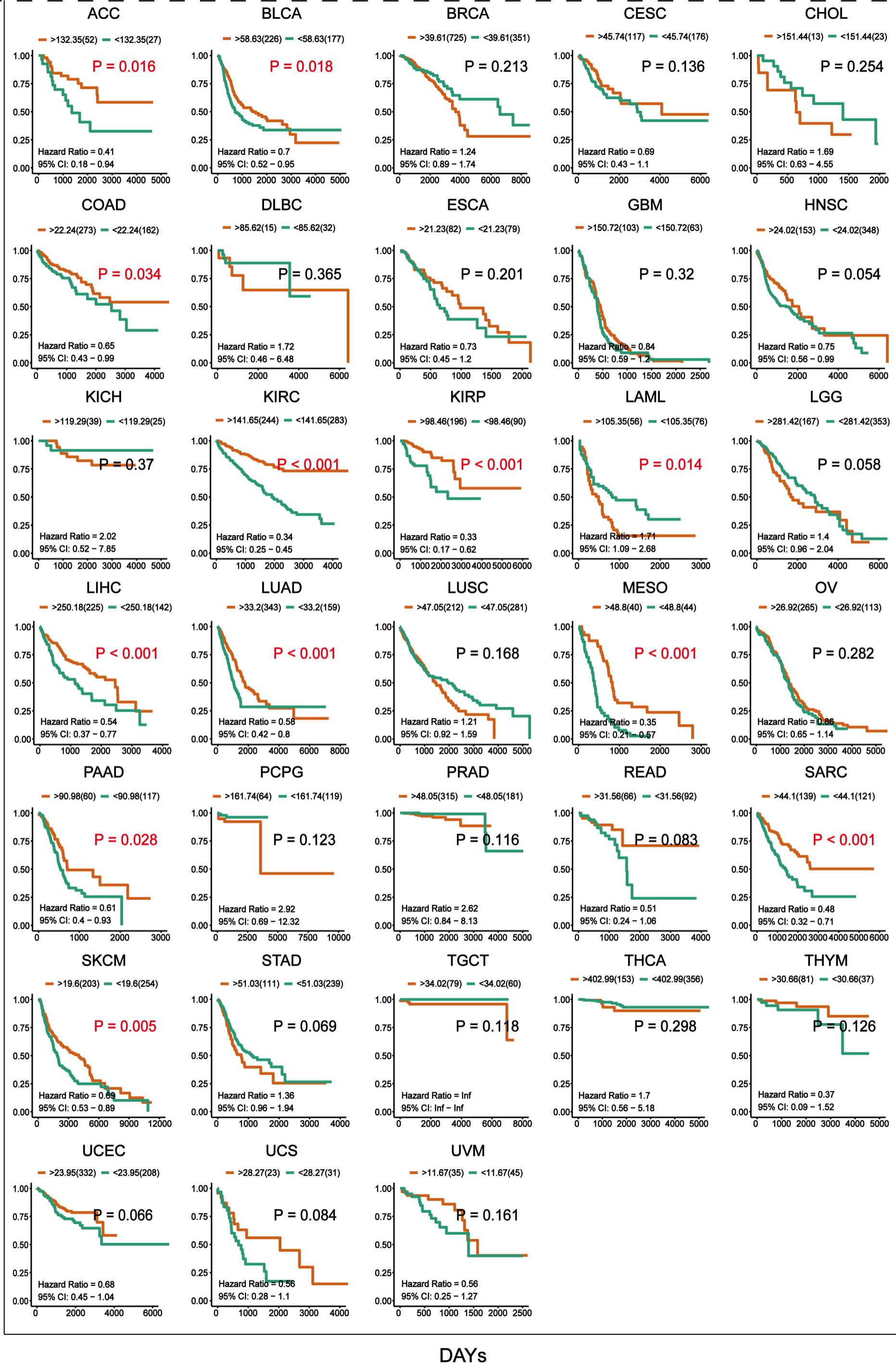

Supplement: Supplemental Information 1 [file peerj-11-15799-s001.pdf]

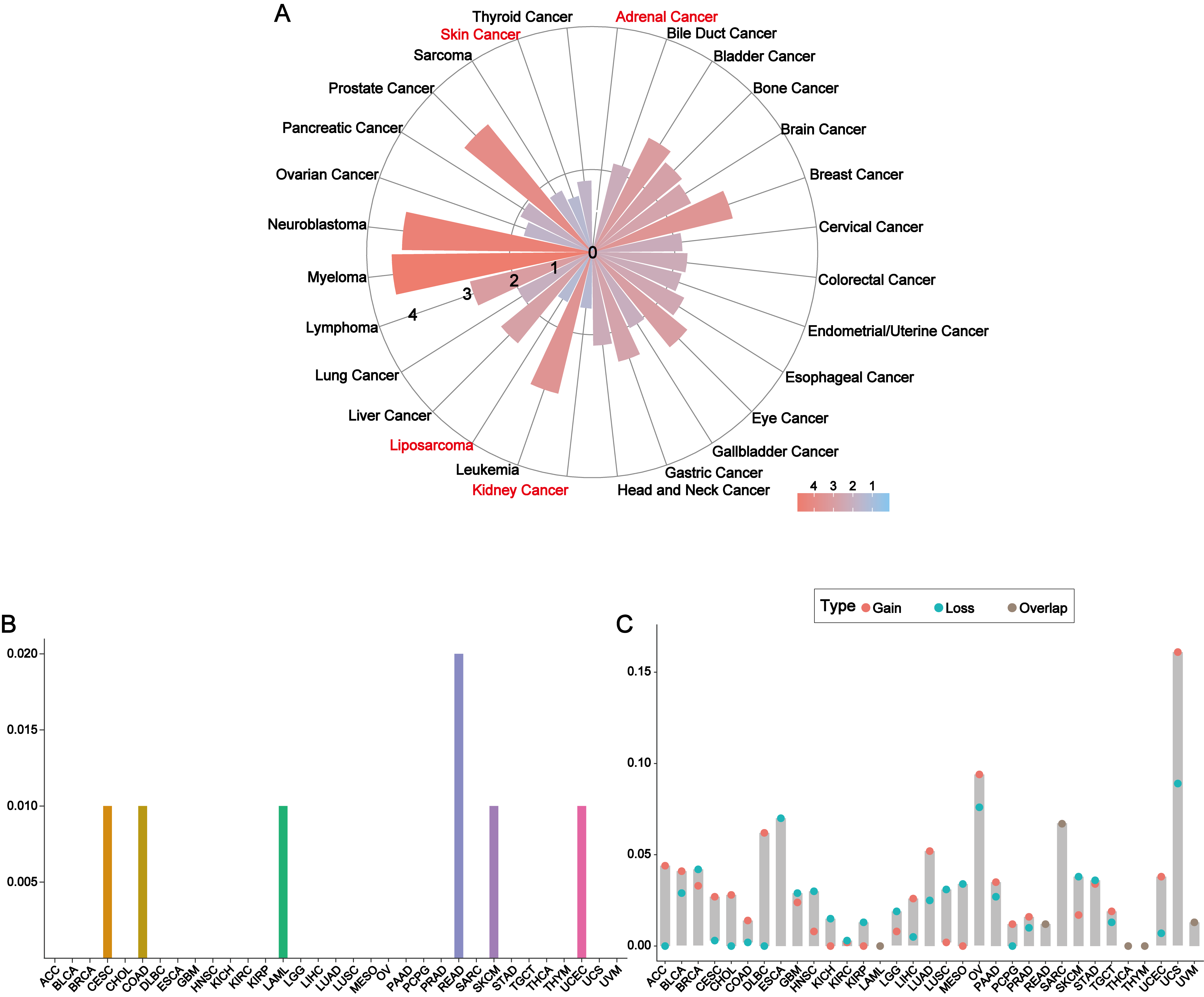

Supplement: Supplemental Information 2 — (A) The expression level of METTL7A in different tumor cell lines. (B) Mutations of METTL7A in different cancer types. (C) Copy number variation of METTL7A in different cancer types. [file peerj-11-15799-s002.png]

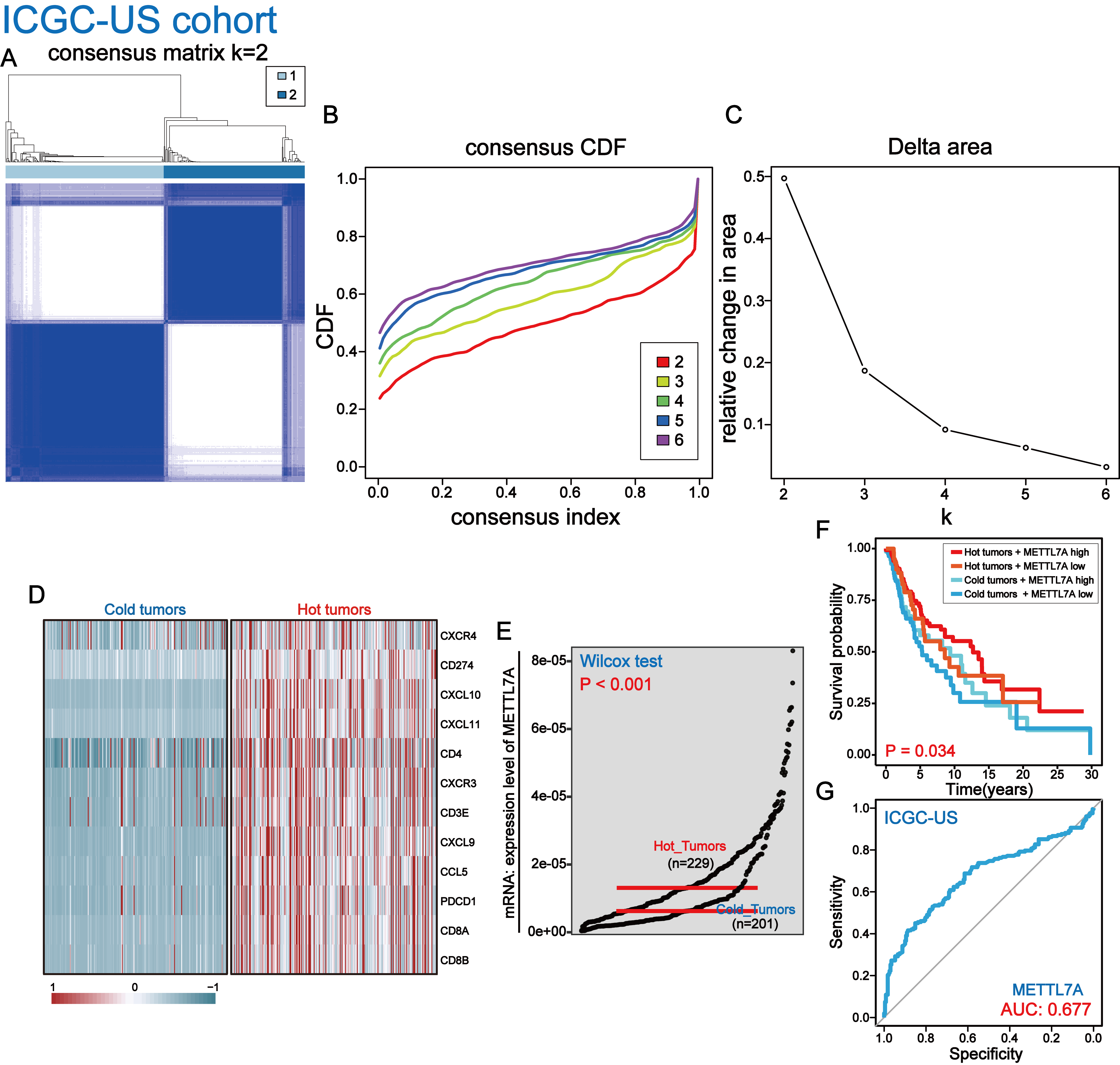

Supplement: Supplemental Information 3 — (A–D) Melanoma patients were divided into hot tumor samples and cold tumor samples by a set of hot tumor signature genes. (E) METTL7A is highly expressed in tumor samples. (F) In METTL7A high expression and hot tumor combined group, patients have the longest overall survival while patients in METTL7A low expression and cold tumor combined group have the lowest overall survival. (G) METTL7A has moderately efficient diagnostic value for hot and cold tumor samples. [file peerj-11-15799-s003.png]

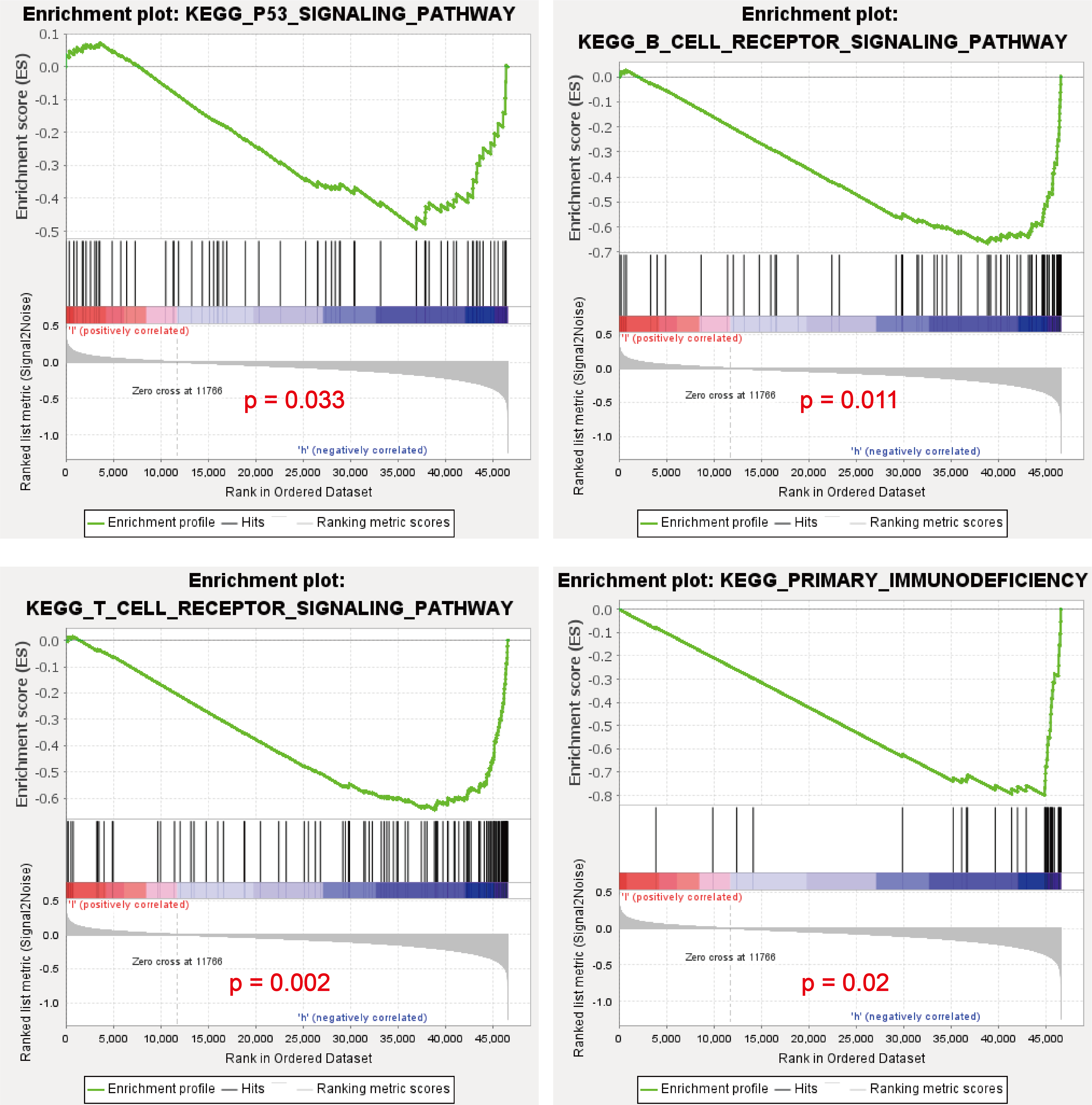

Supplement: Supplemental Information 4 [file peerj-11-15799-s004.png]
