# Supplementary material for: Transcriptomic characterization revealed that METTL7A inhibits melanoma progression via the p53 signaling pathway and immunomodulatory pathway
Source: PeerJ. 2023 Aug 2;11:e15799. doi: 10.7717/peerj.15799 (PMC10404031; doi:10.7717/peerj.15799)
Supplement: Supplemental Information 7 [file peerj-11-15799-s007.zip › Uncropped_western_blots_Figure_5c.docx]

**Original uncropped images of western blots used for the Figure 5c.**

**SKMEL28**

**A2058**


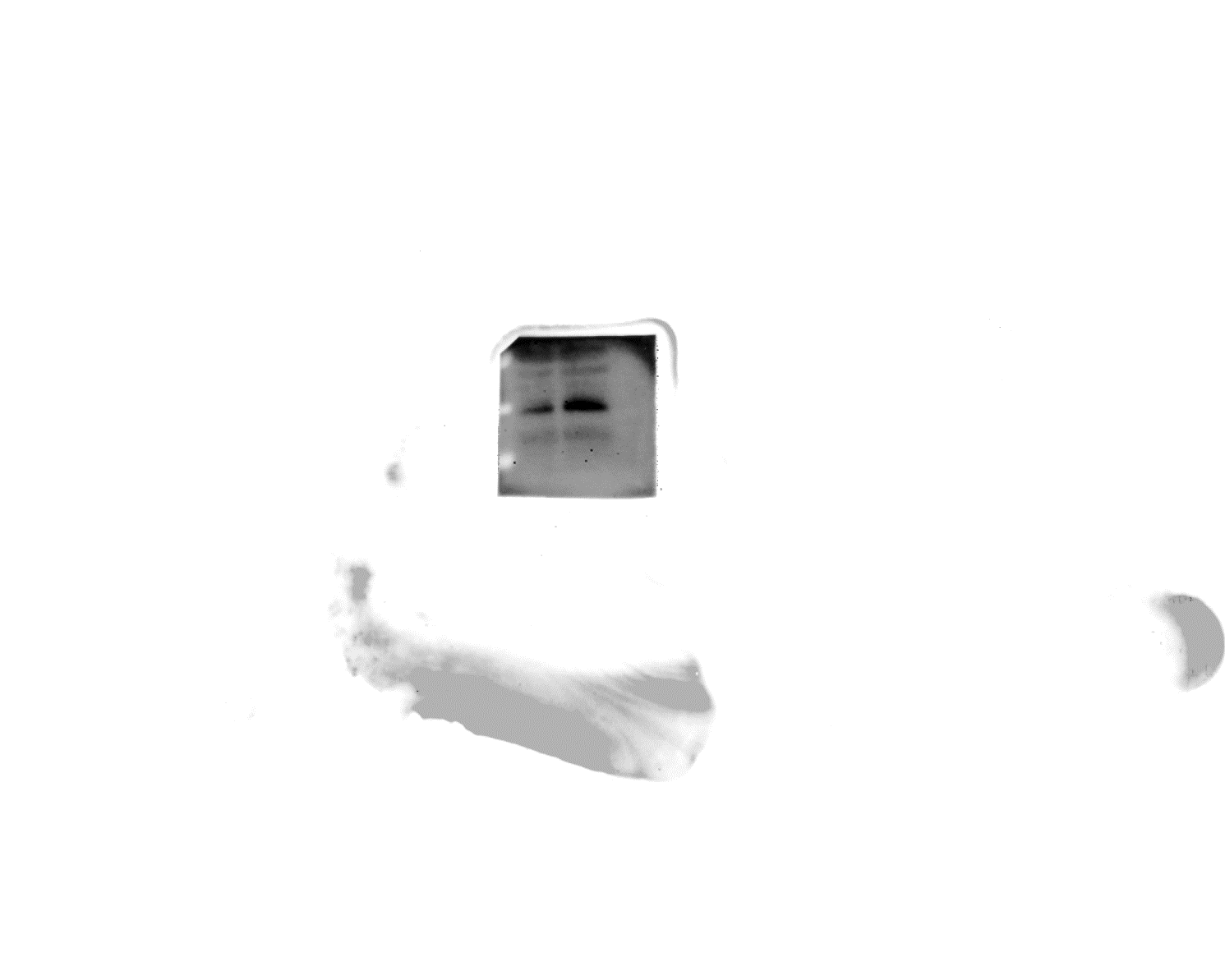

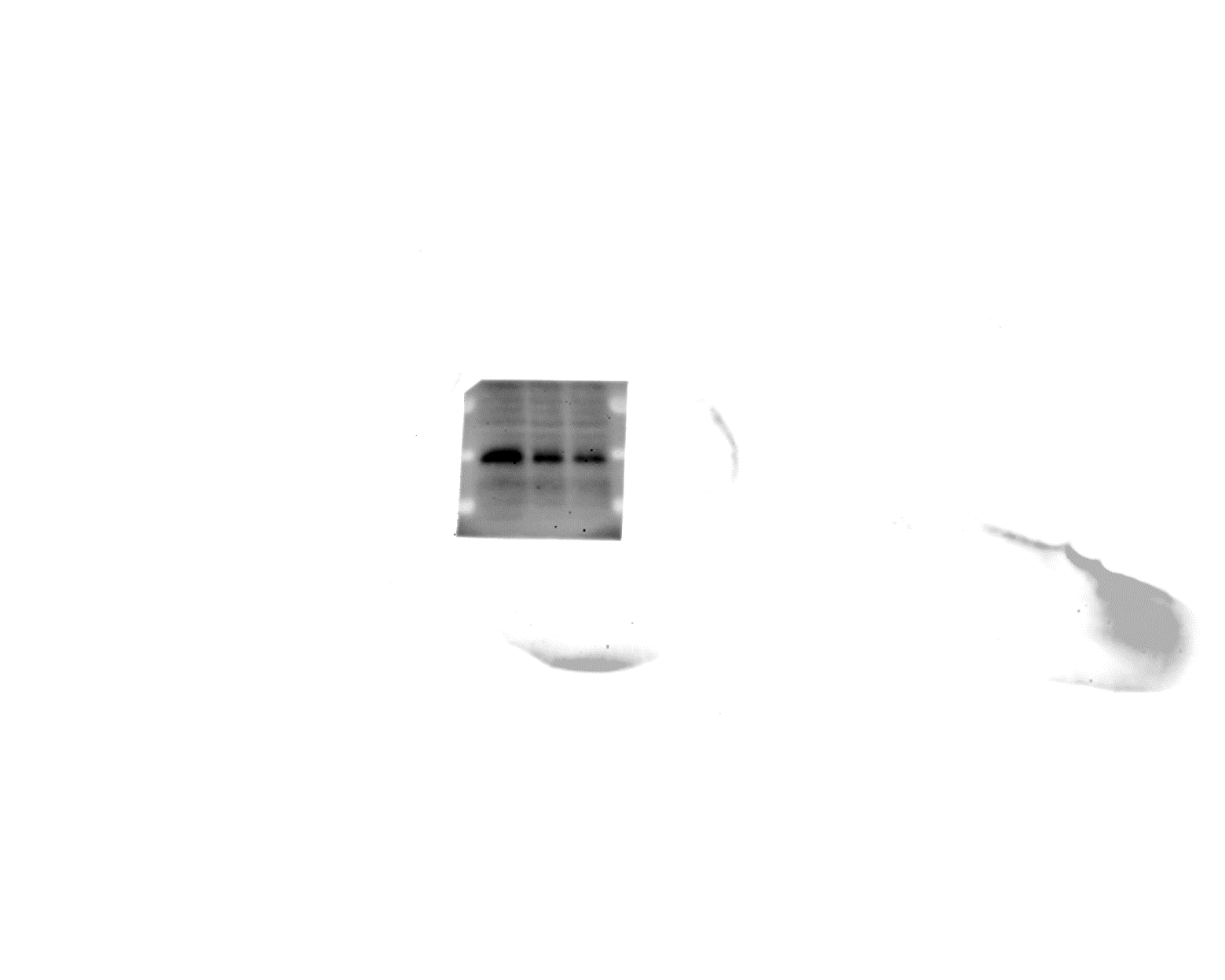

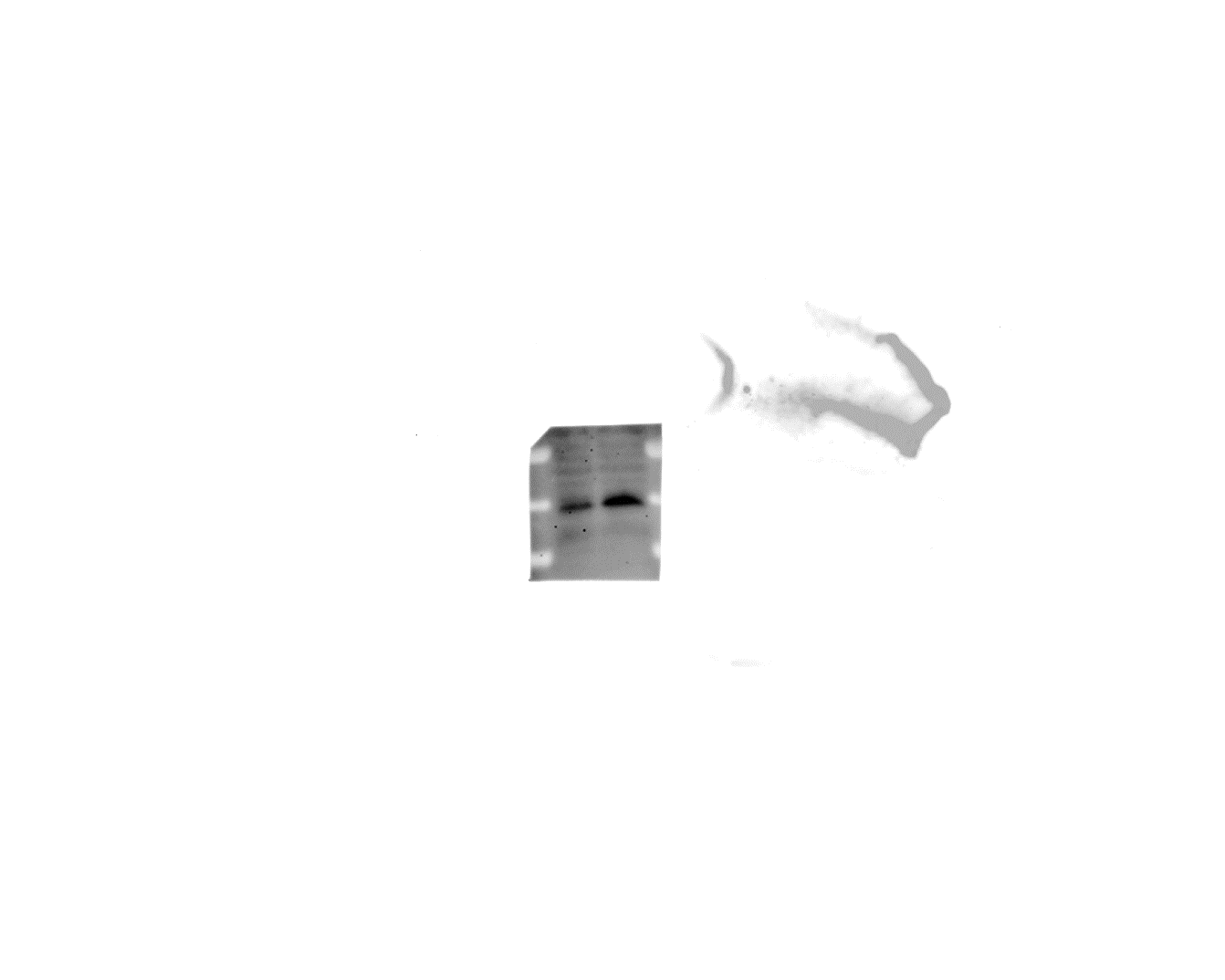

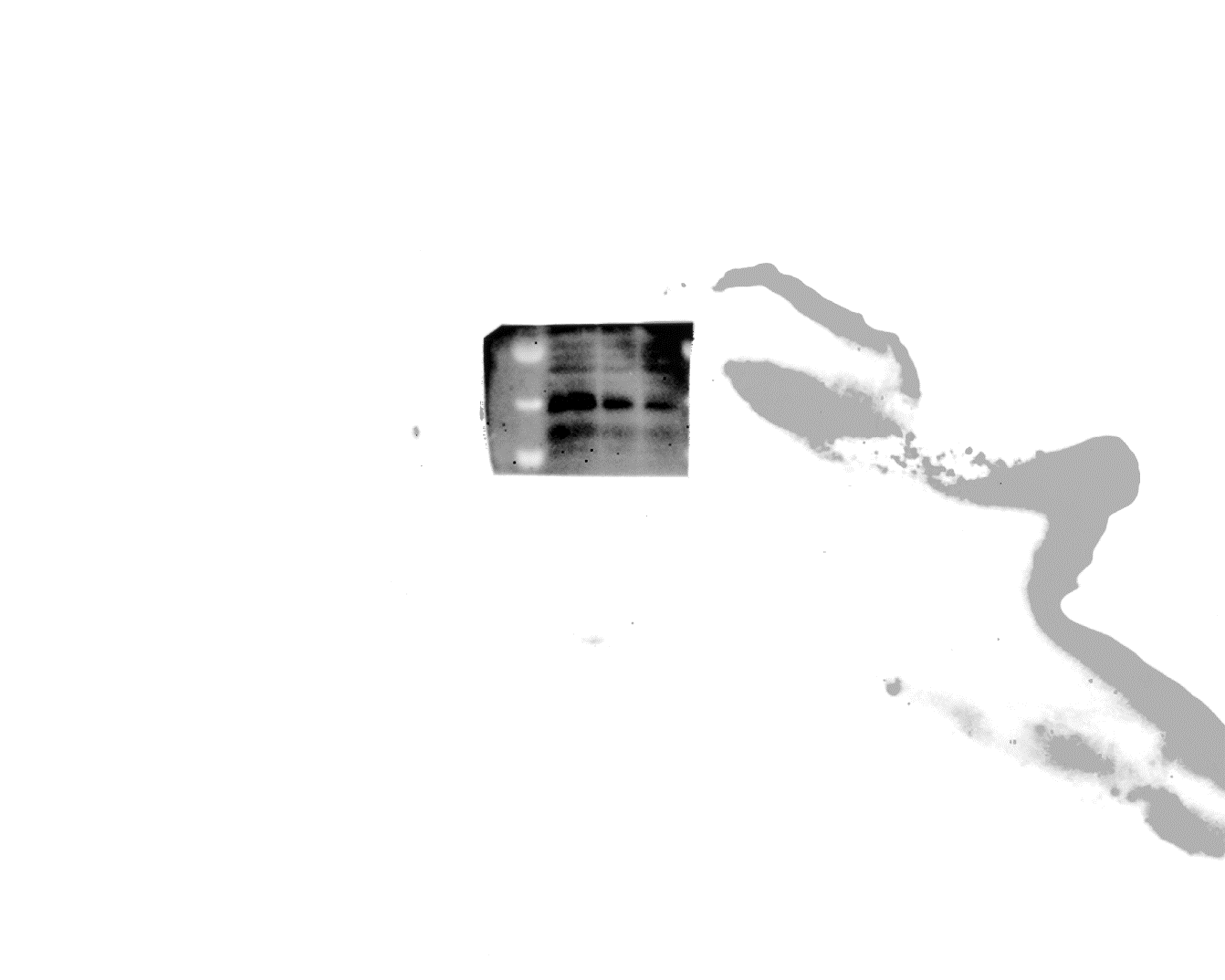

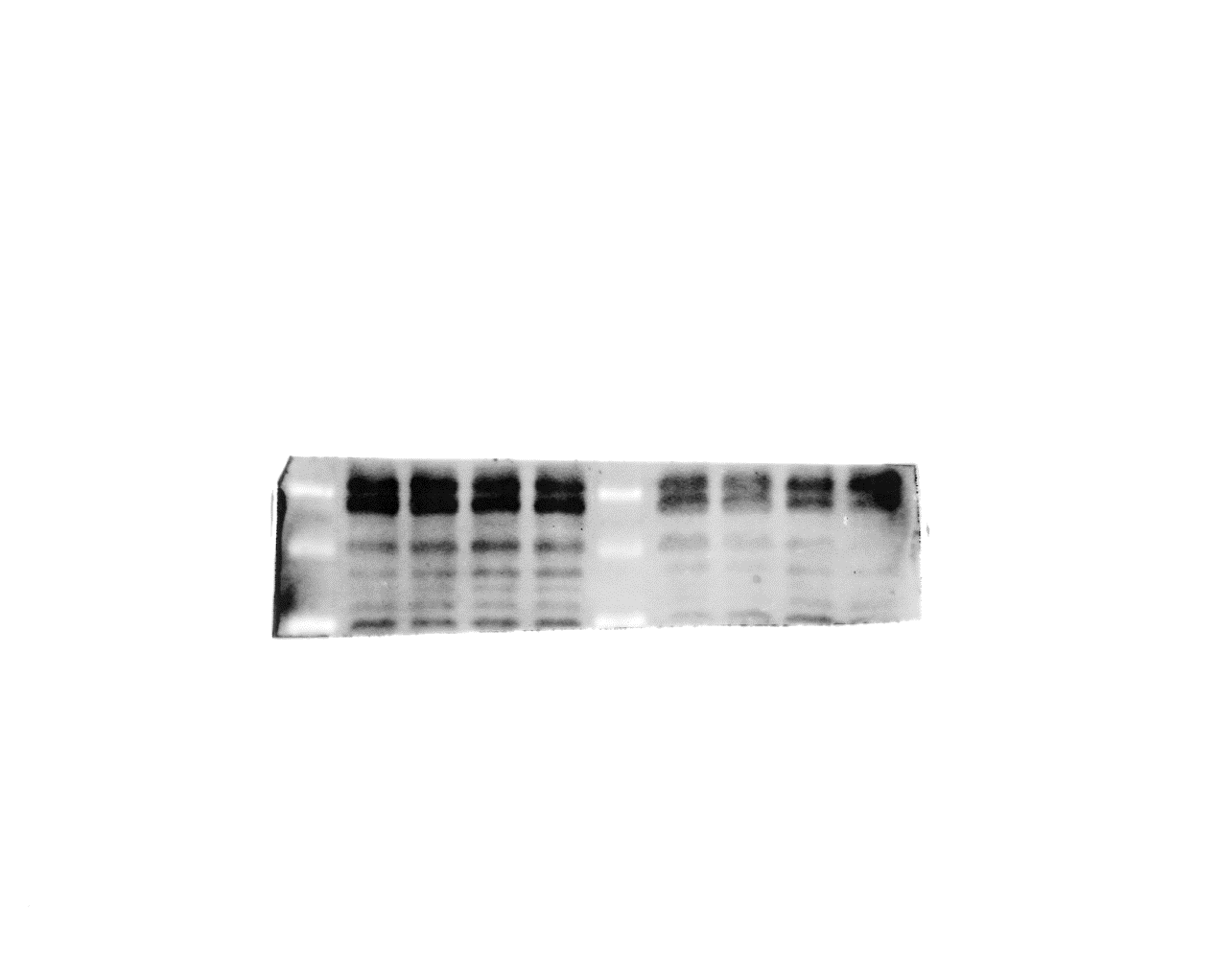

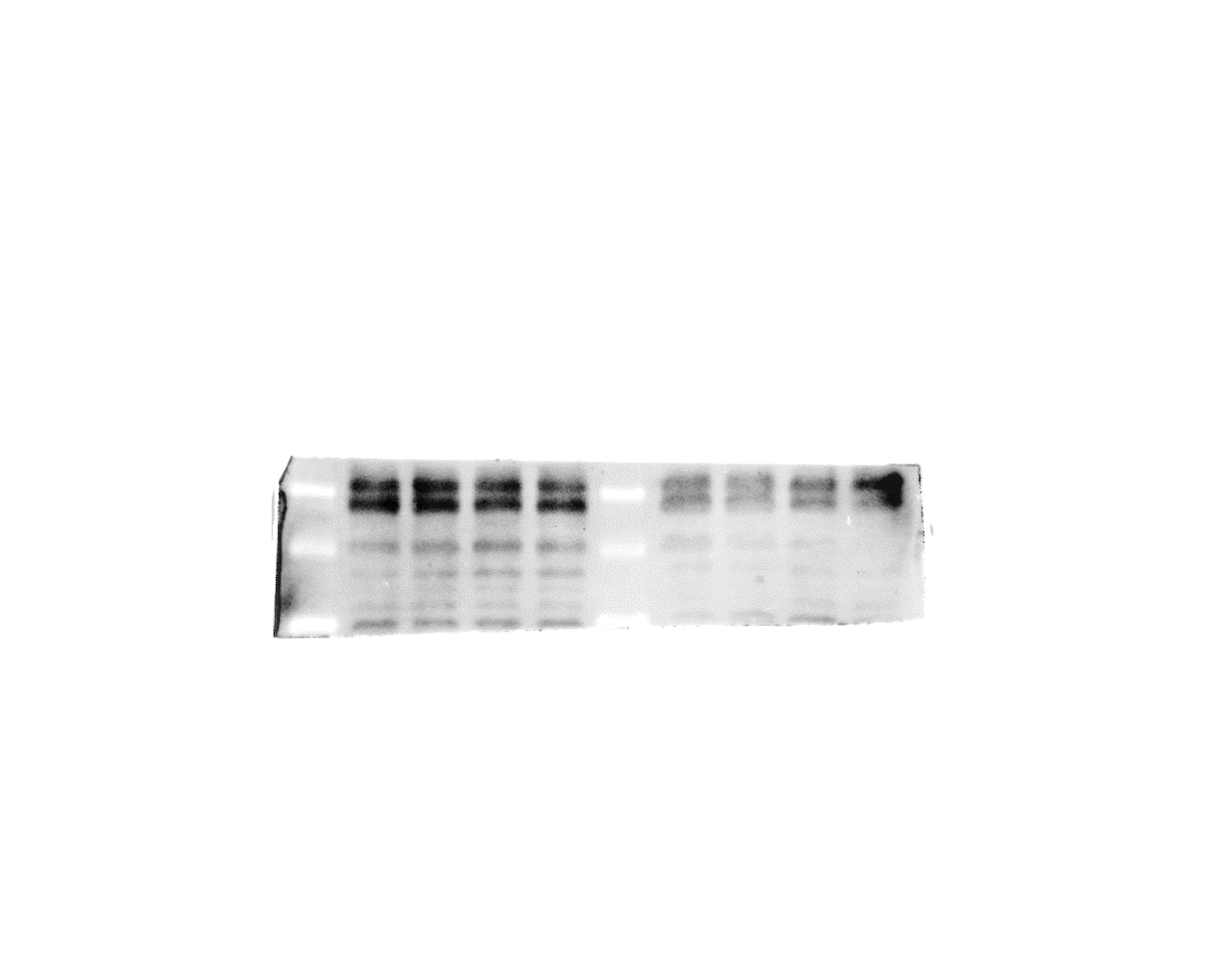

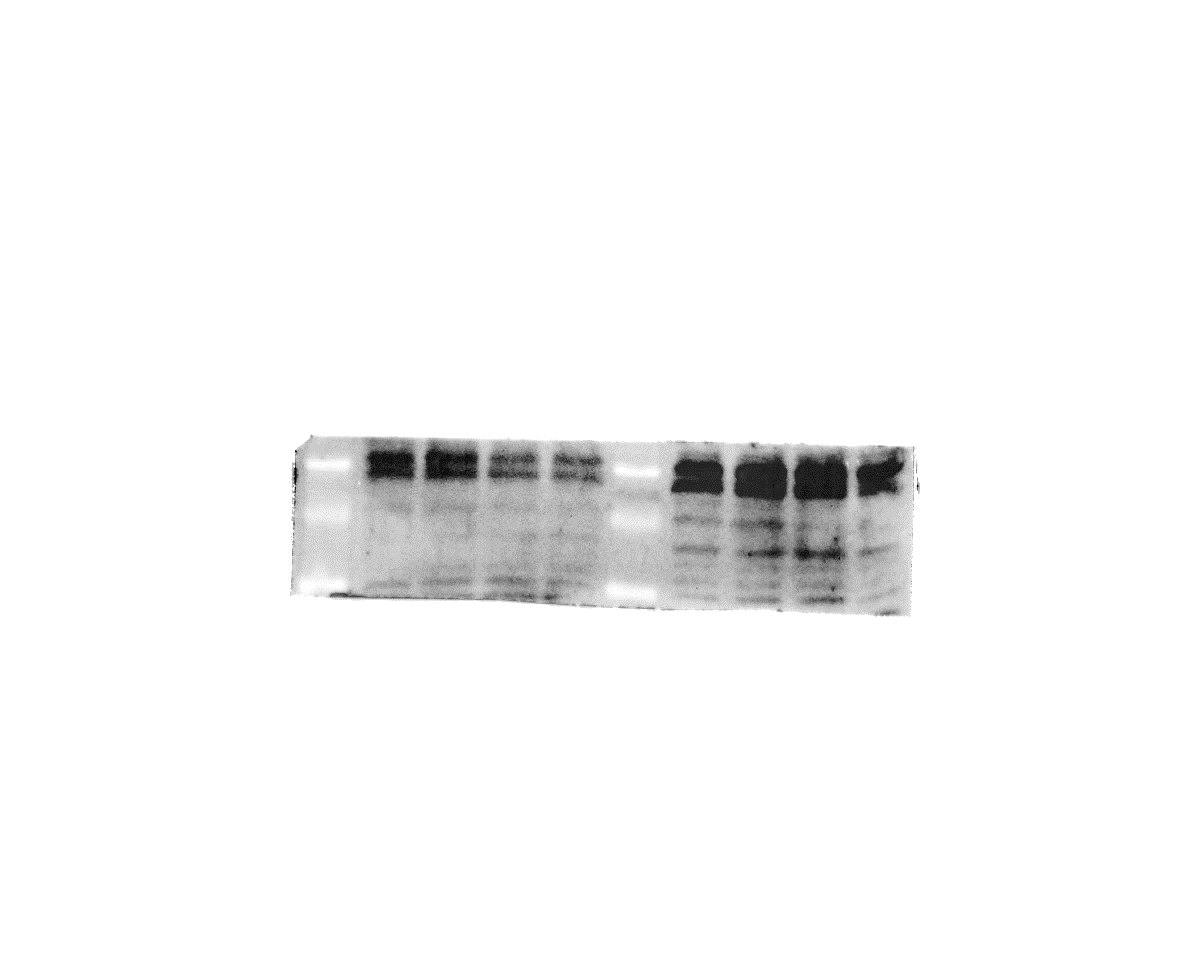

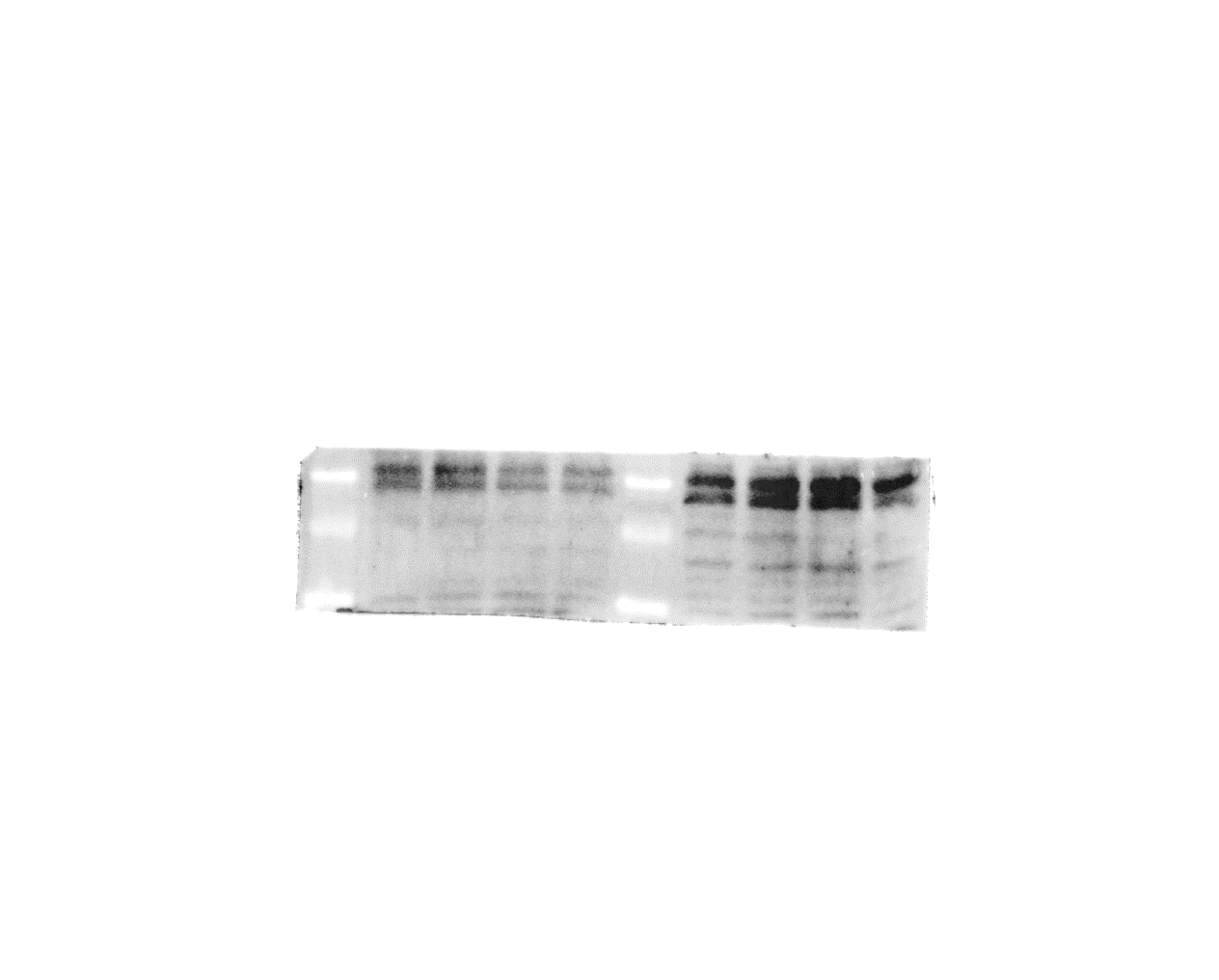

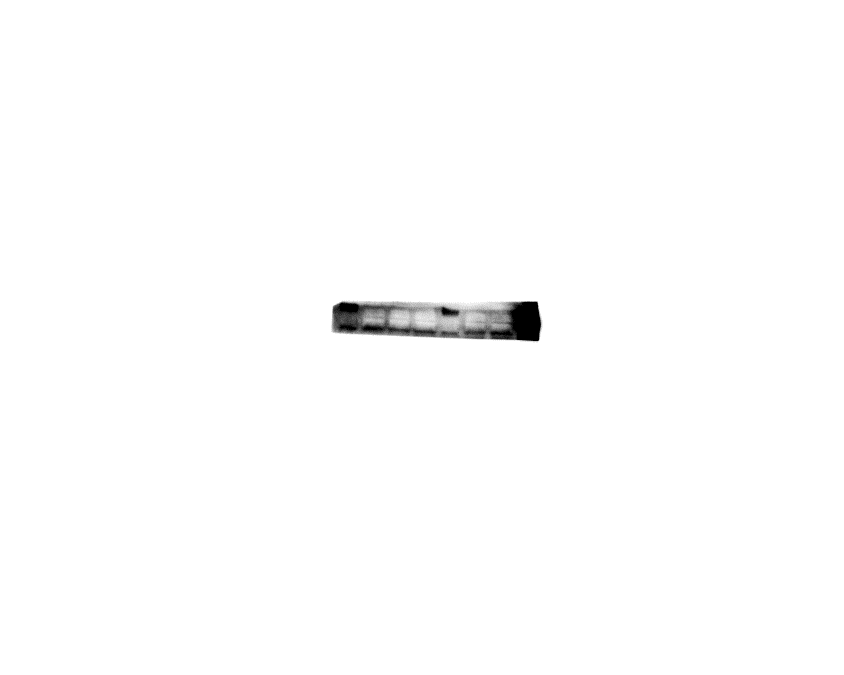

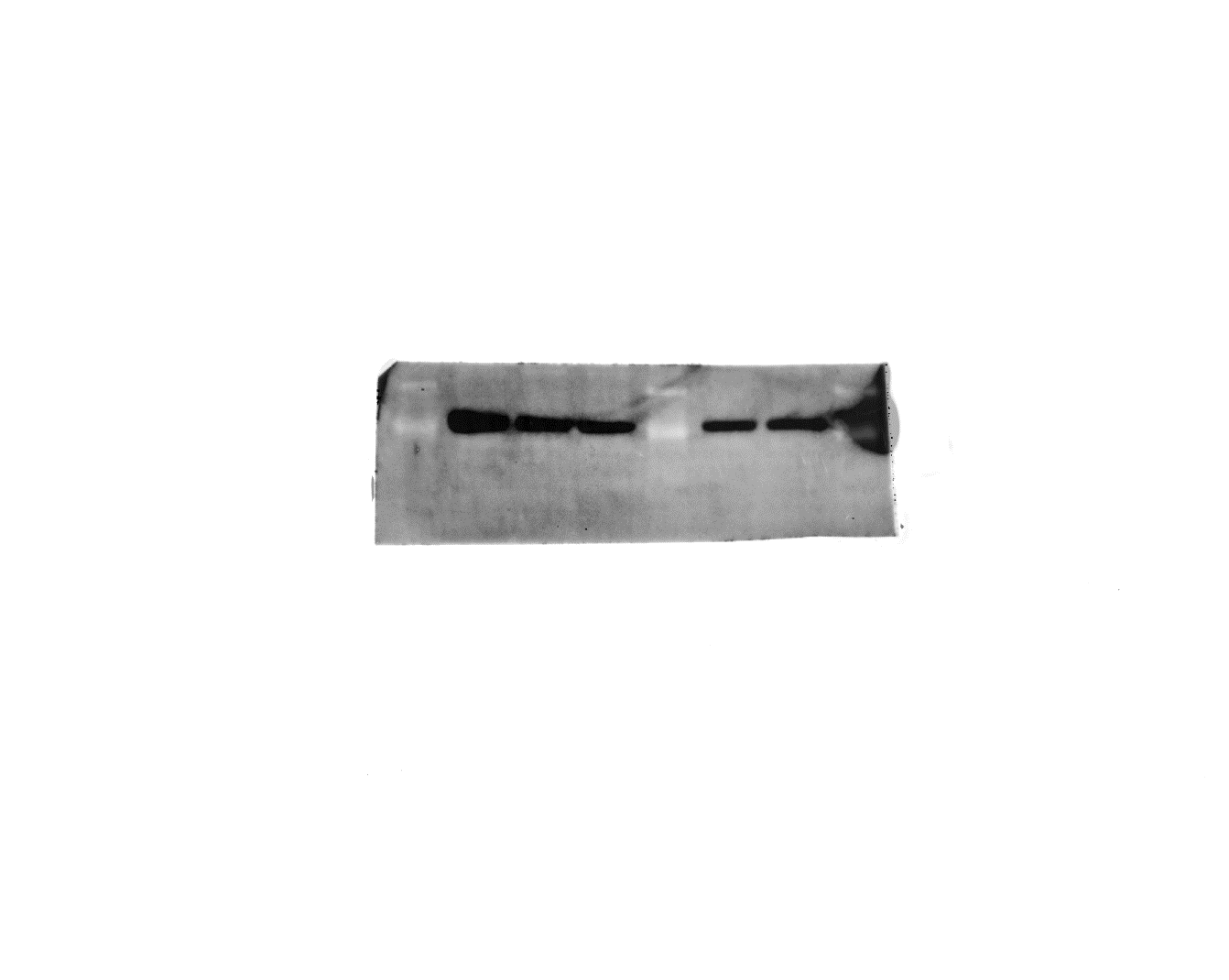

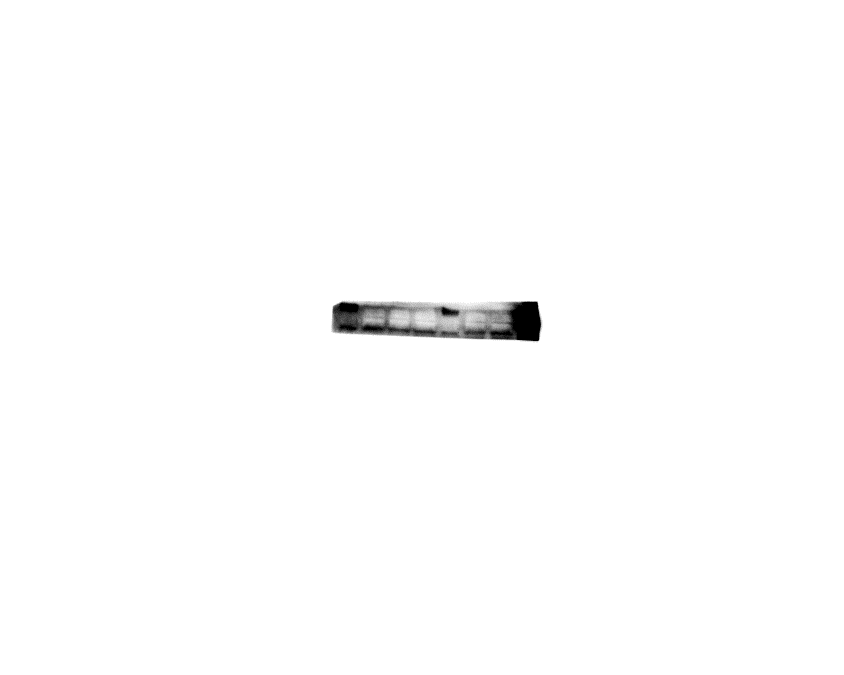

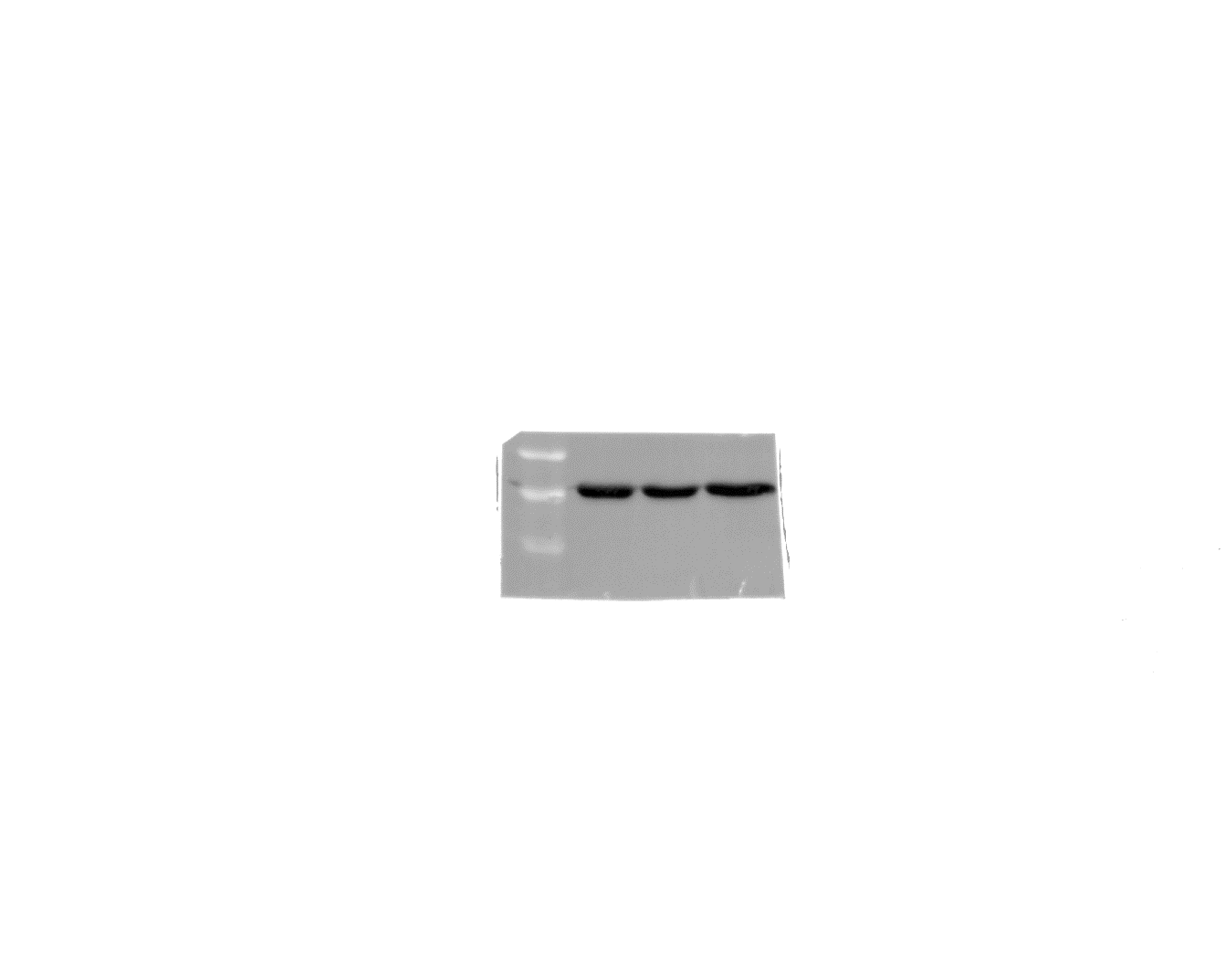

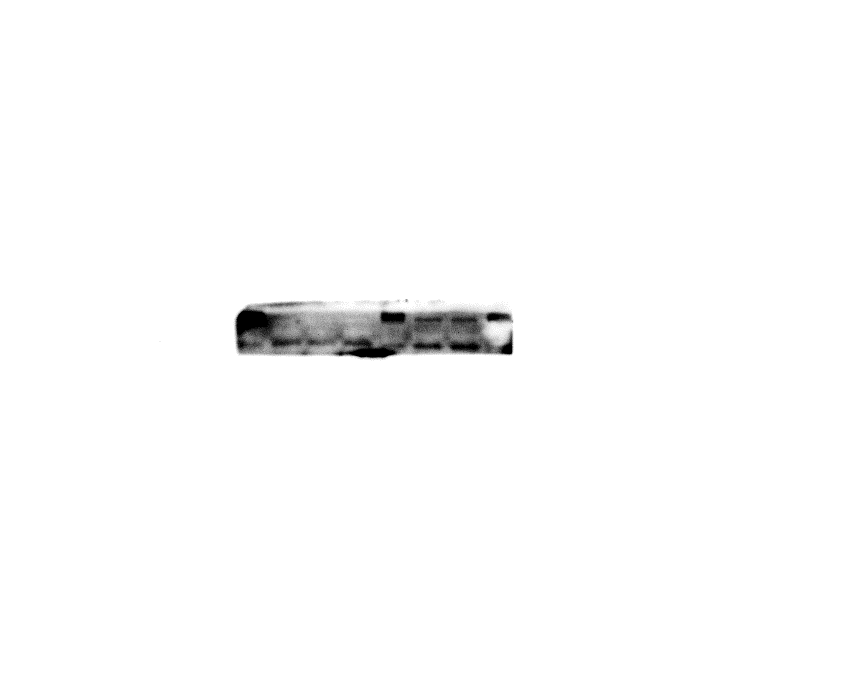

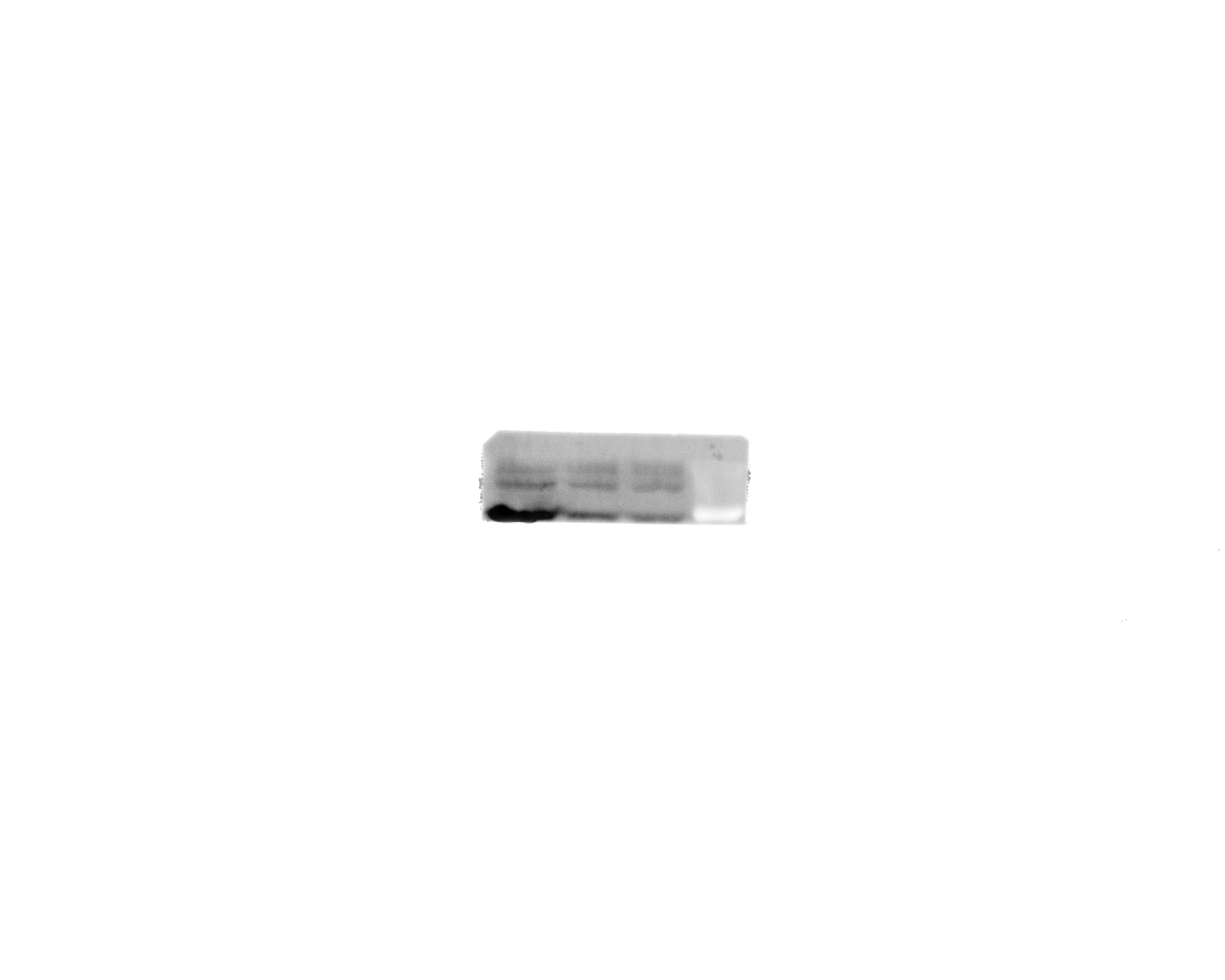

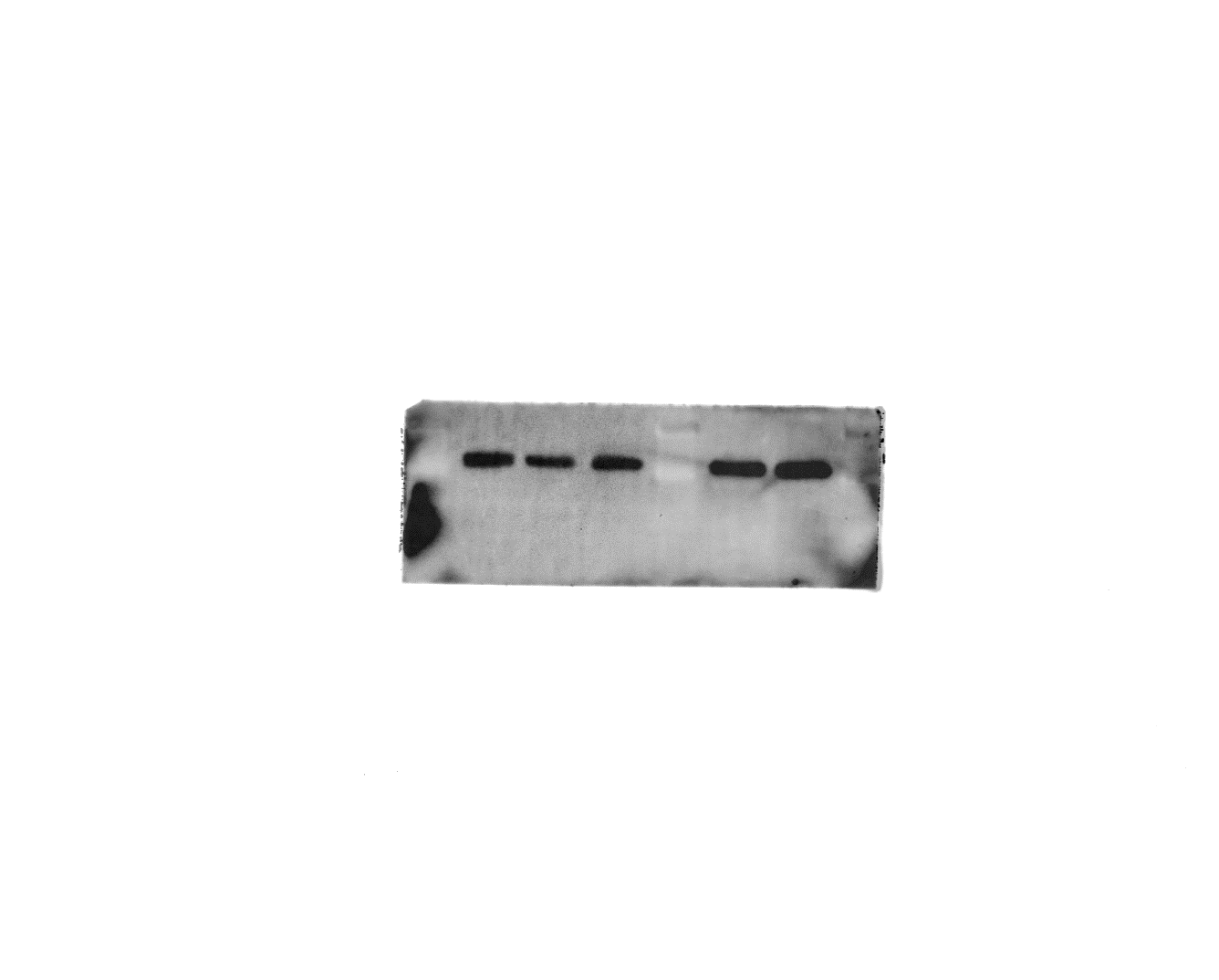

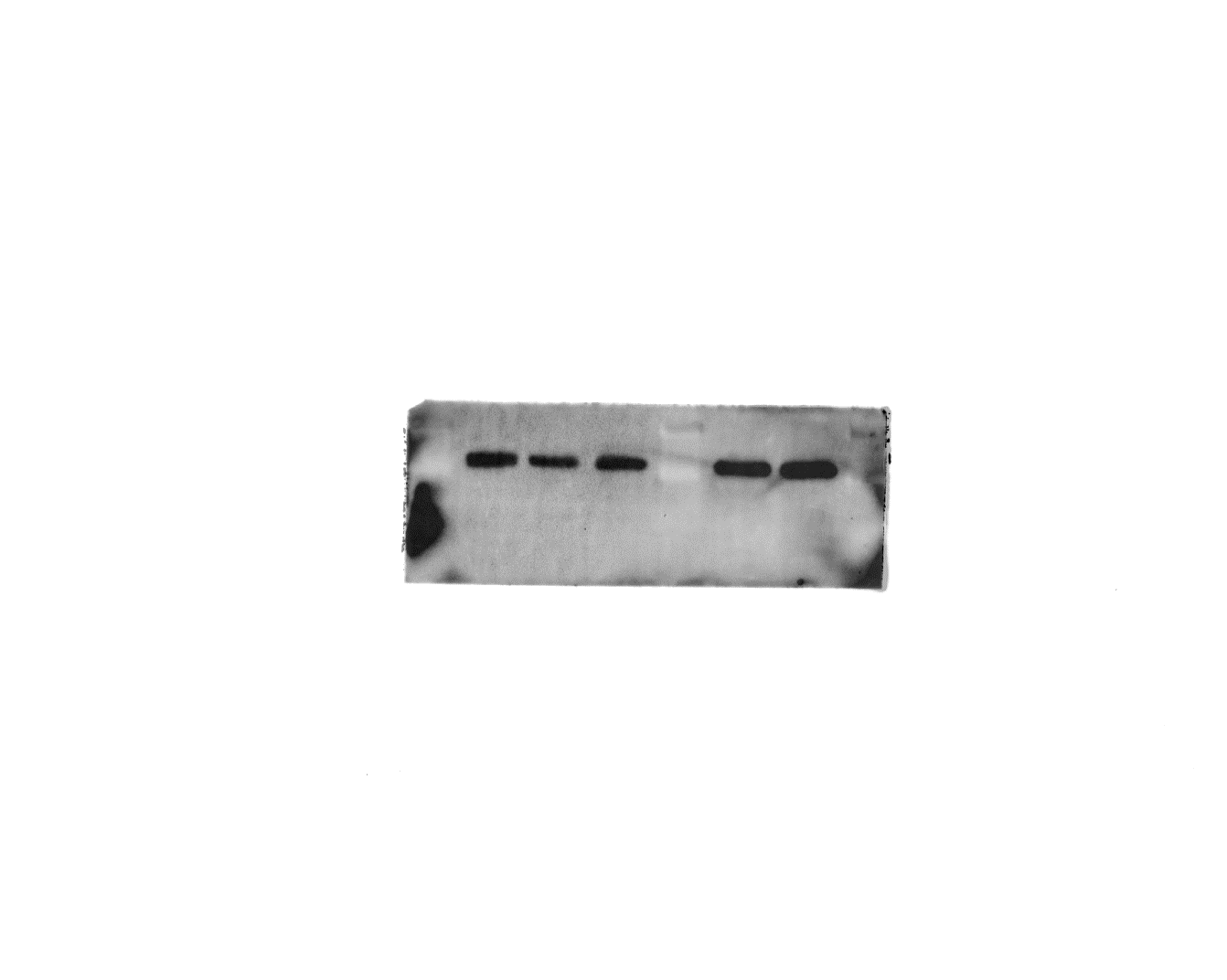


M

siMETTL7A-2

siMETTL7A-1

METTL7A

Control

M

Control

siMETTL7A-2

P21

27KDa

21KDa

53KDa

37KDa

GAPDH

p53

METTL7A

M

Control

M

M

M

M

M

M

M

M

M

M

M

M

siMETTL7A-1

siMETTL7A-1

siMETTL7A-1

Control

Control

Control

Control

Control

Control

siMETTL7A-2

siMETTL7A-2

siMETTL7A-2

siMETTL7A-2

METTL7A

METTL7A

METTL7A

METTL7A

METTL7A

METTL7A

METTL7A

siMETTL7A-2

siMETTL7A-2

siMETTL7A-1

siMETTL7A-1

siMETTL7A-1

Control

Control

Control

Control

Control

Control

siMETTL7A-1

Control

siMETTL7A-2

siMETTL7A-1

METTL7A

METTL7A

siMETTL7A-2

Control

Control

Control

siMETTL7A-1

Control


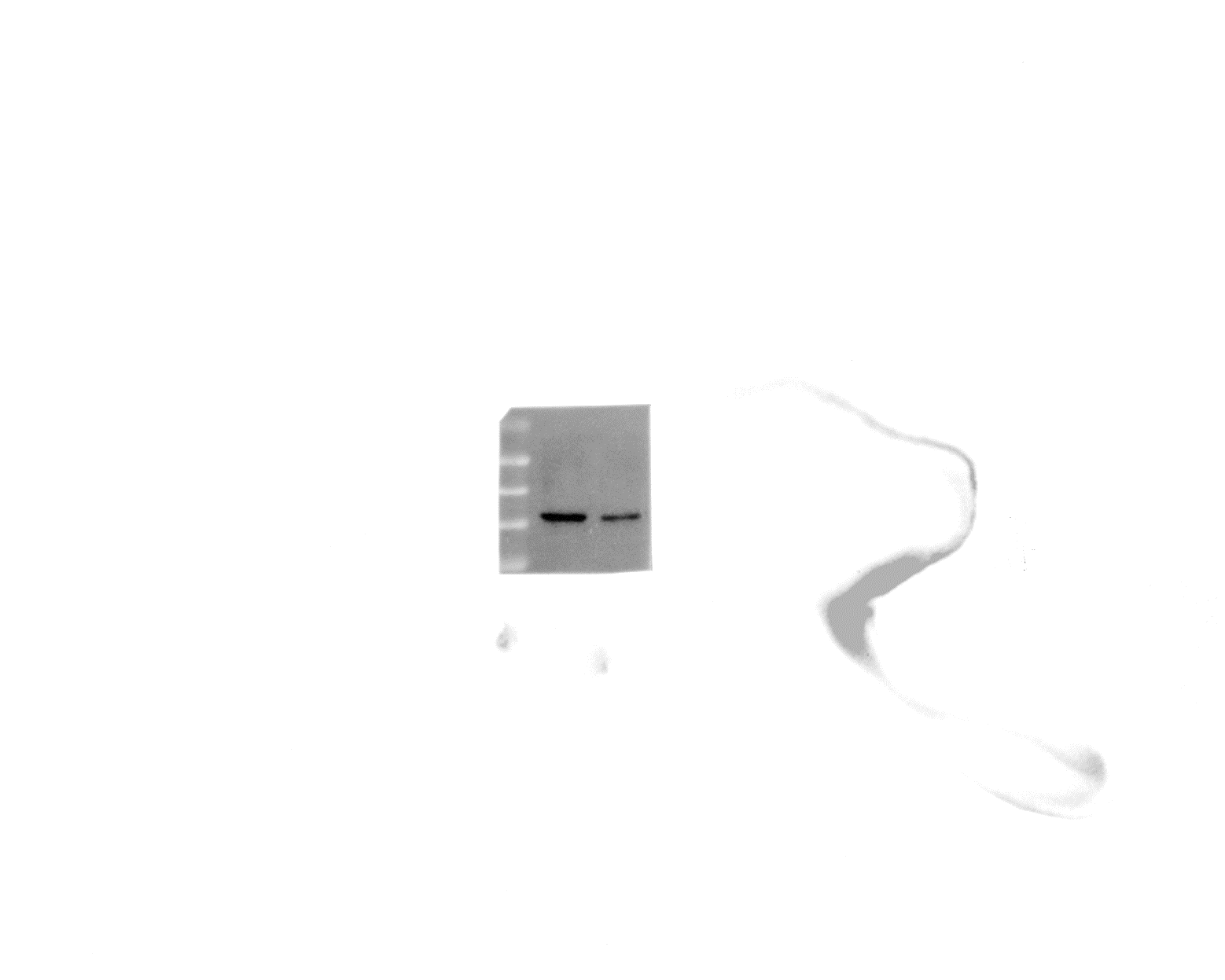

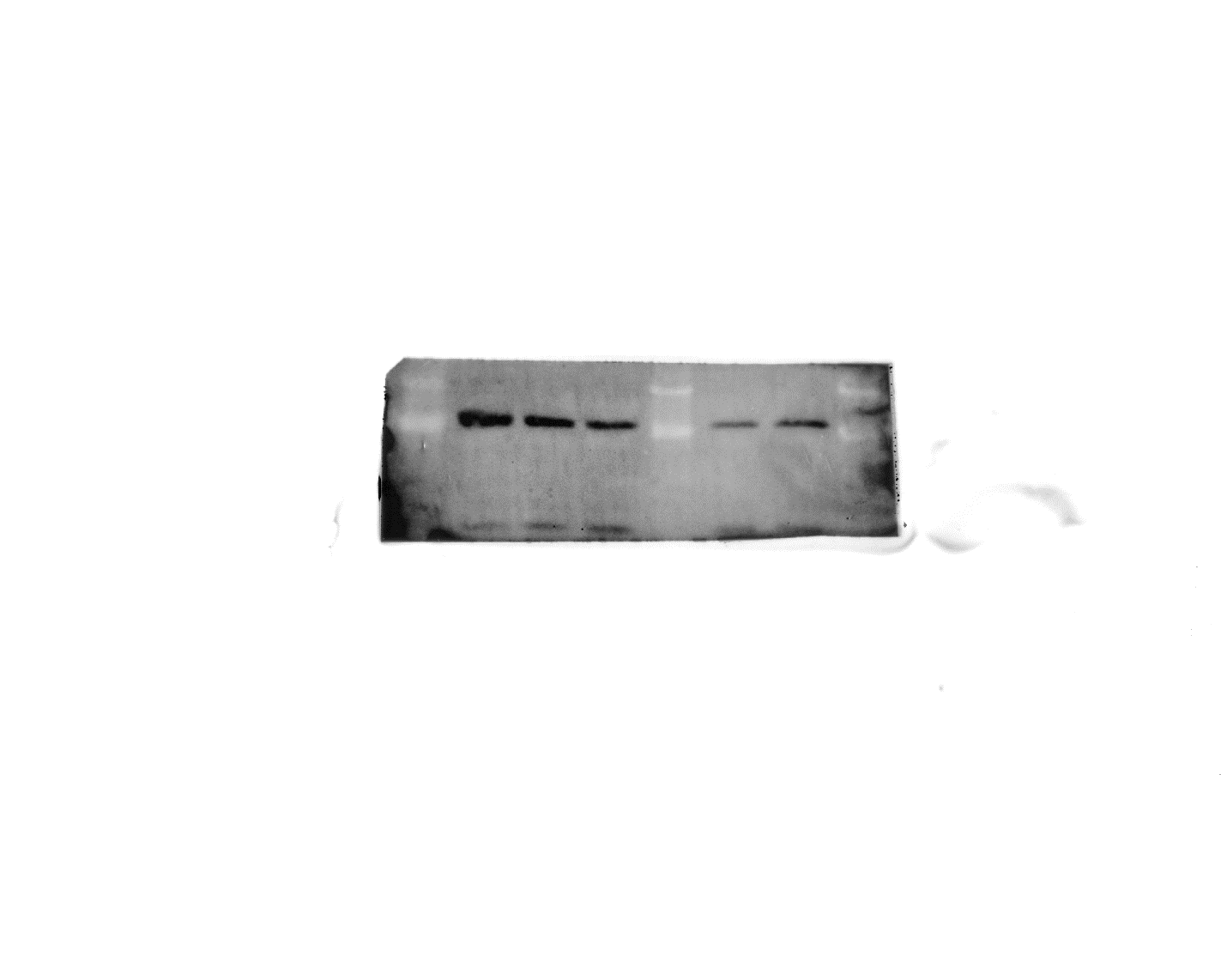

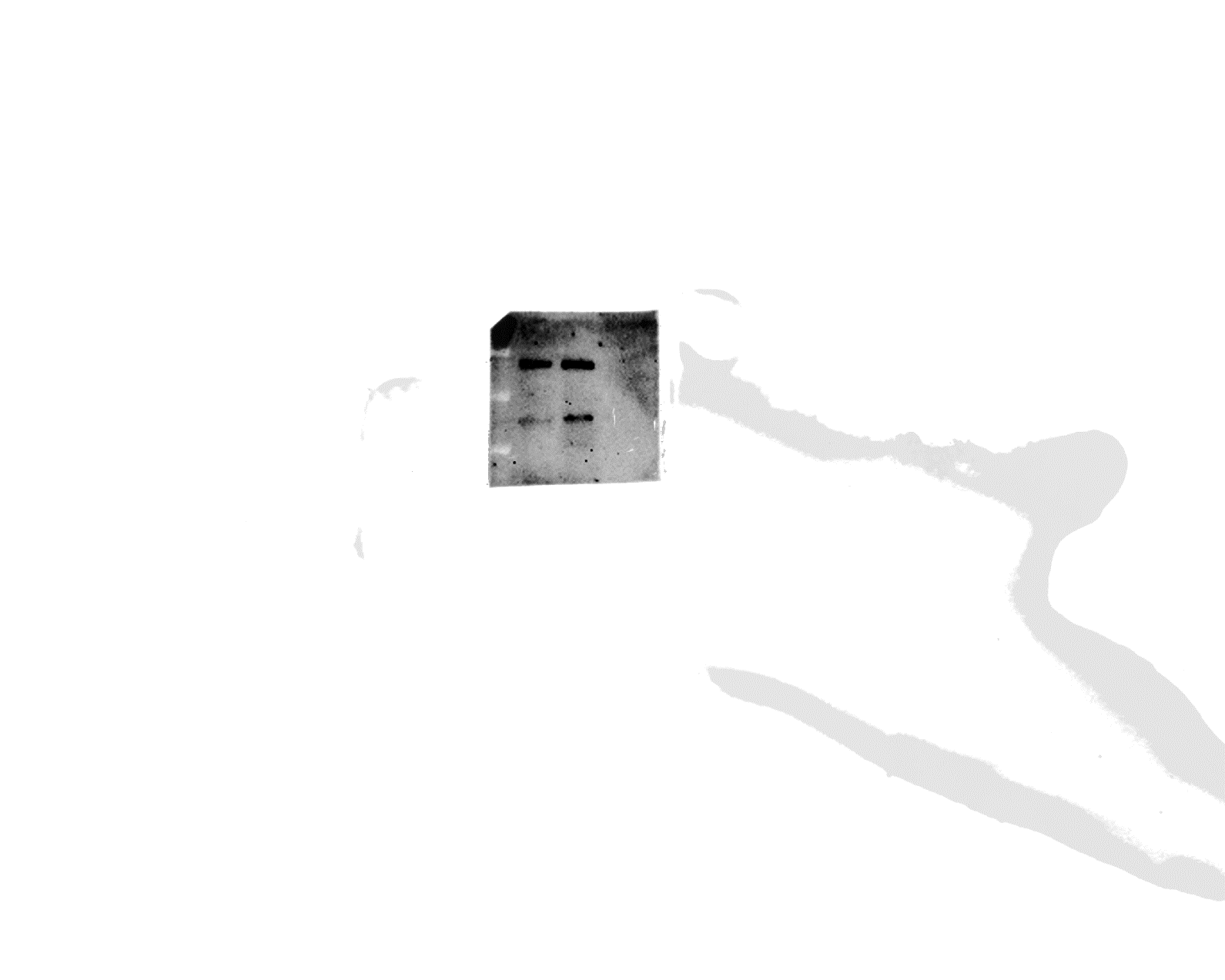

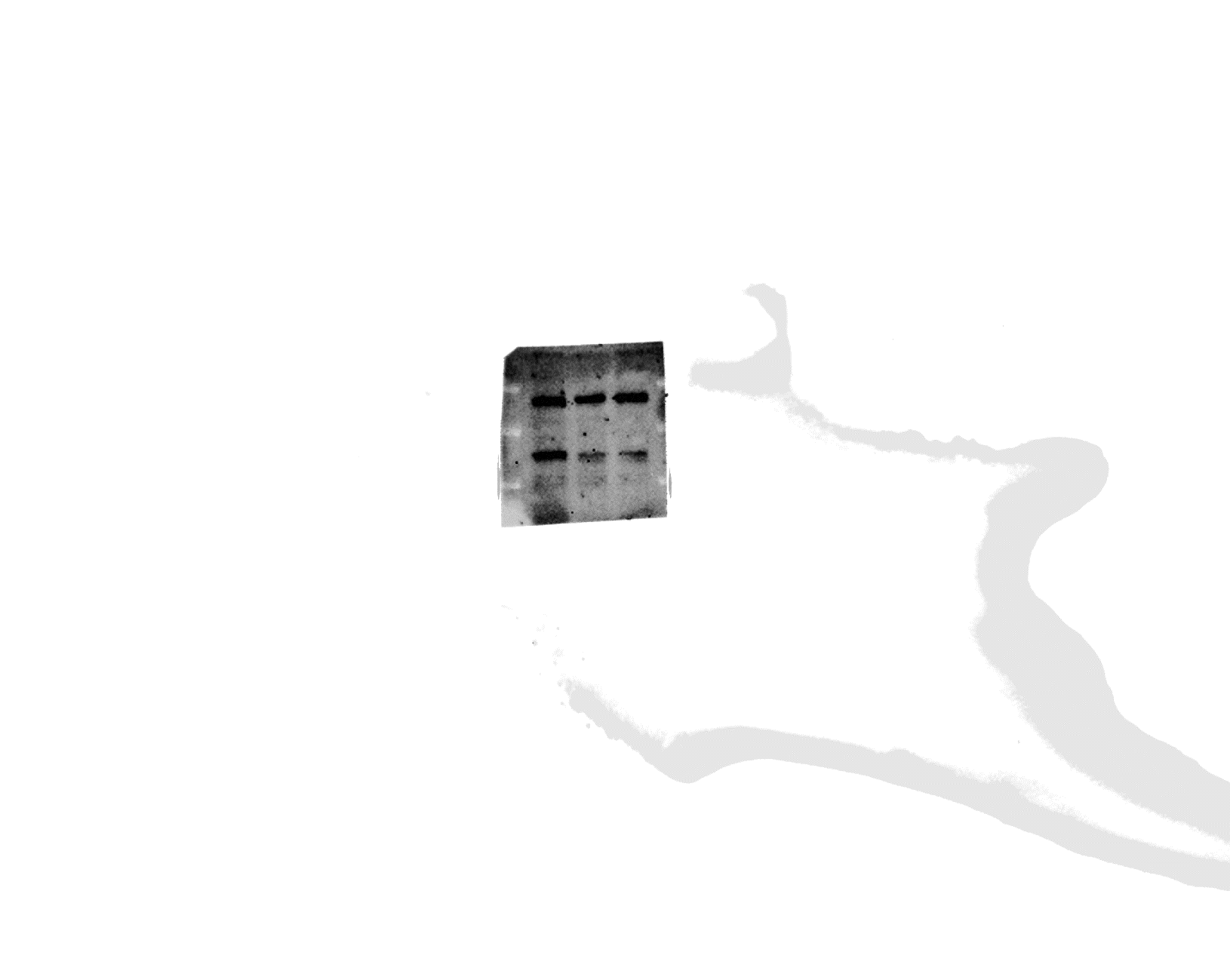

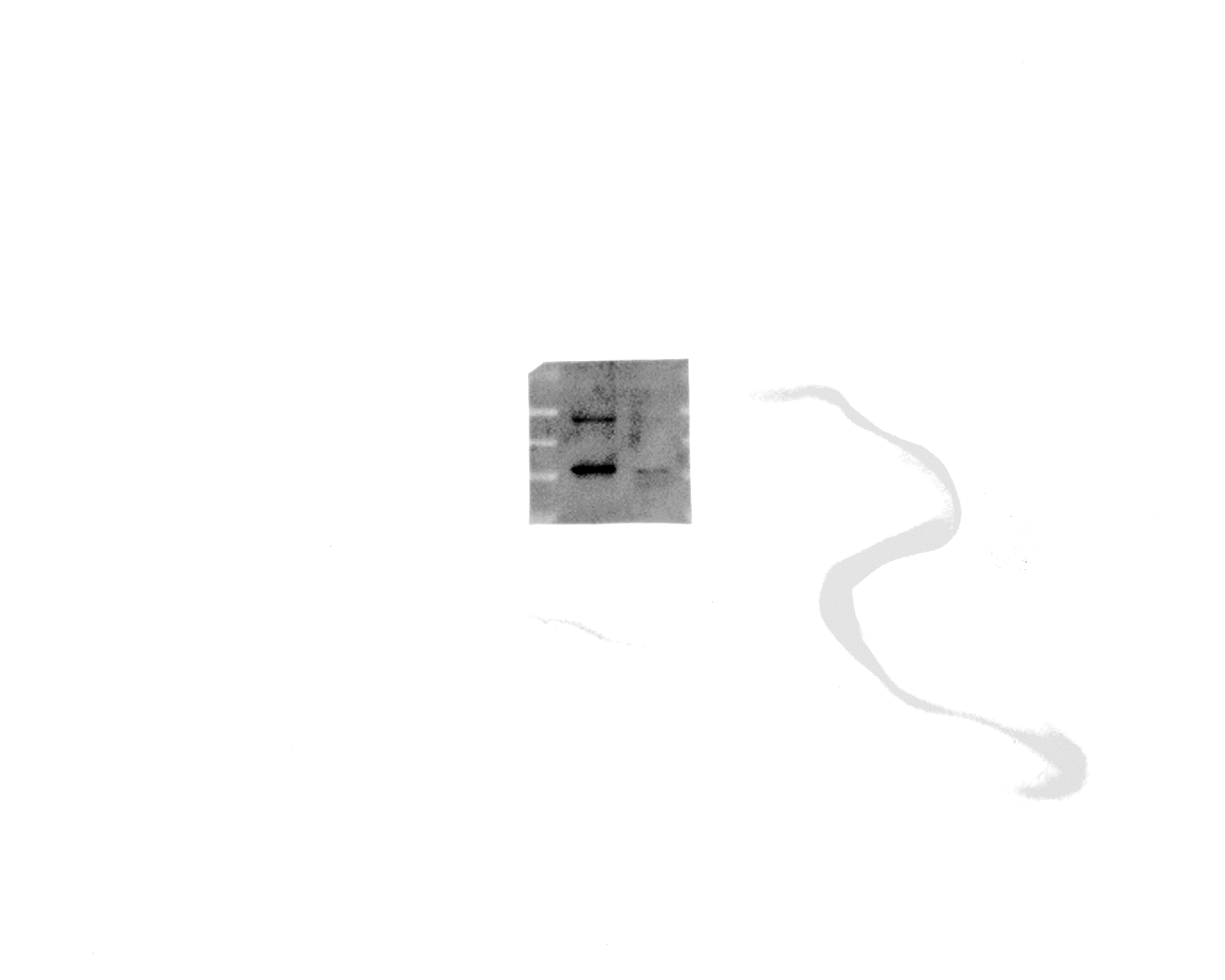

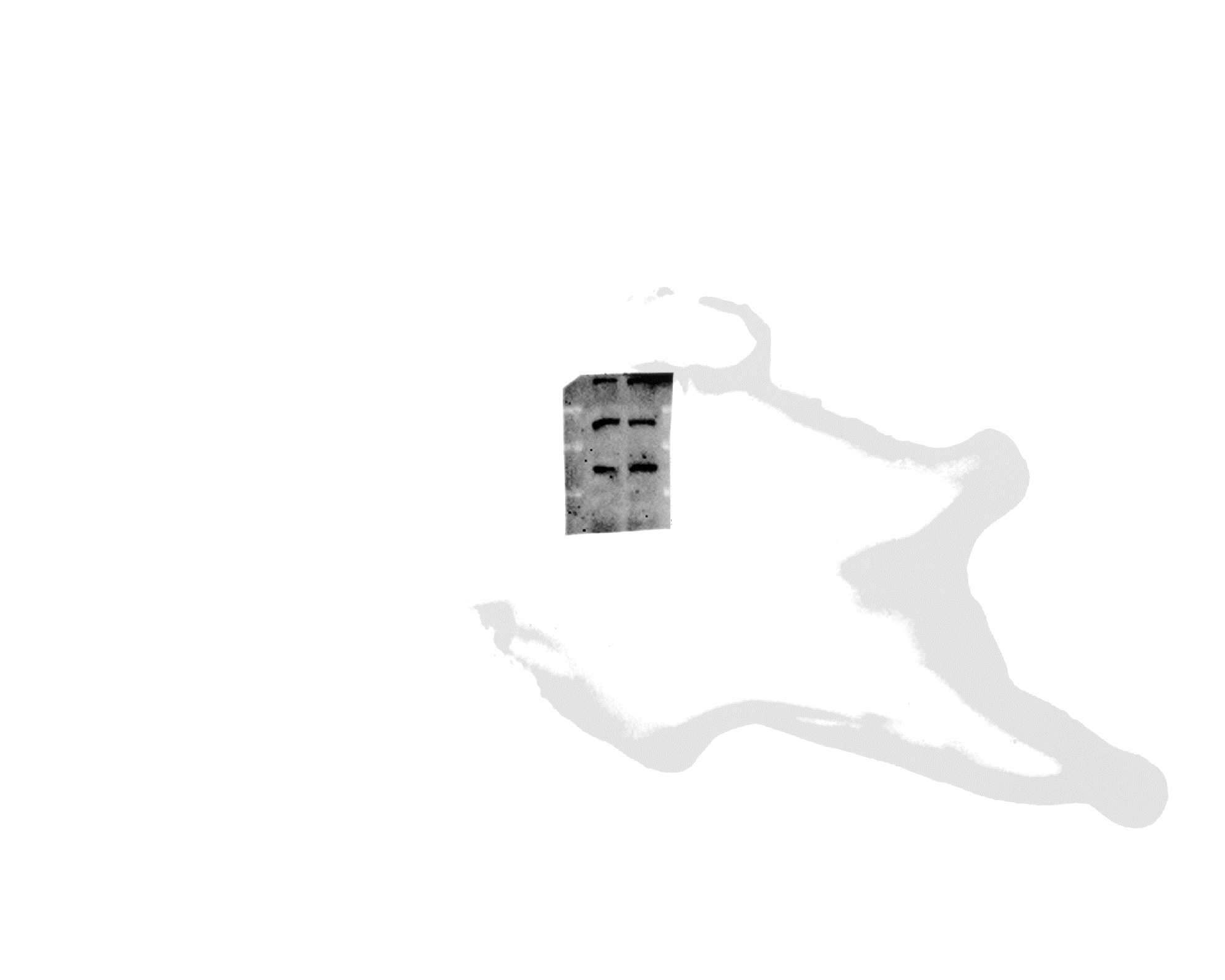

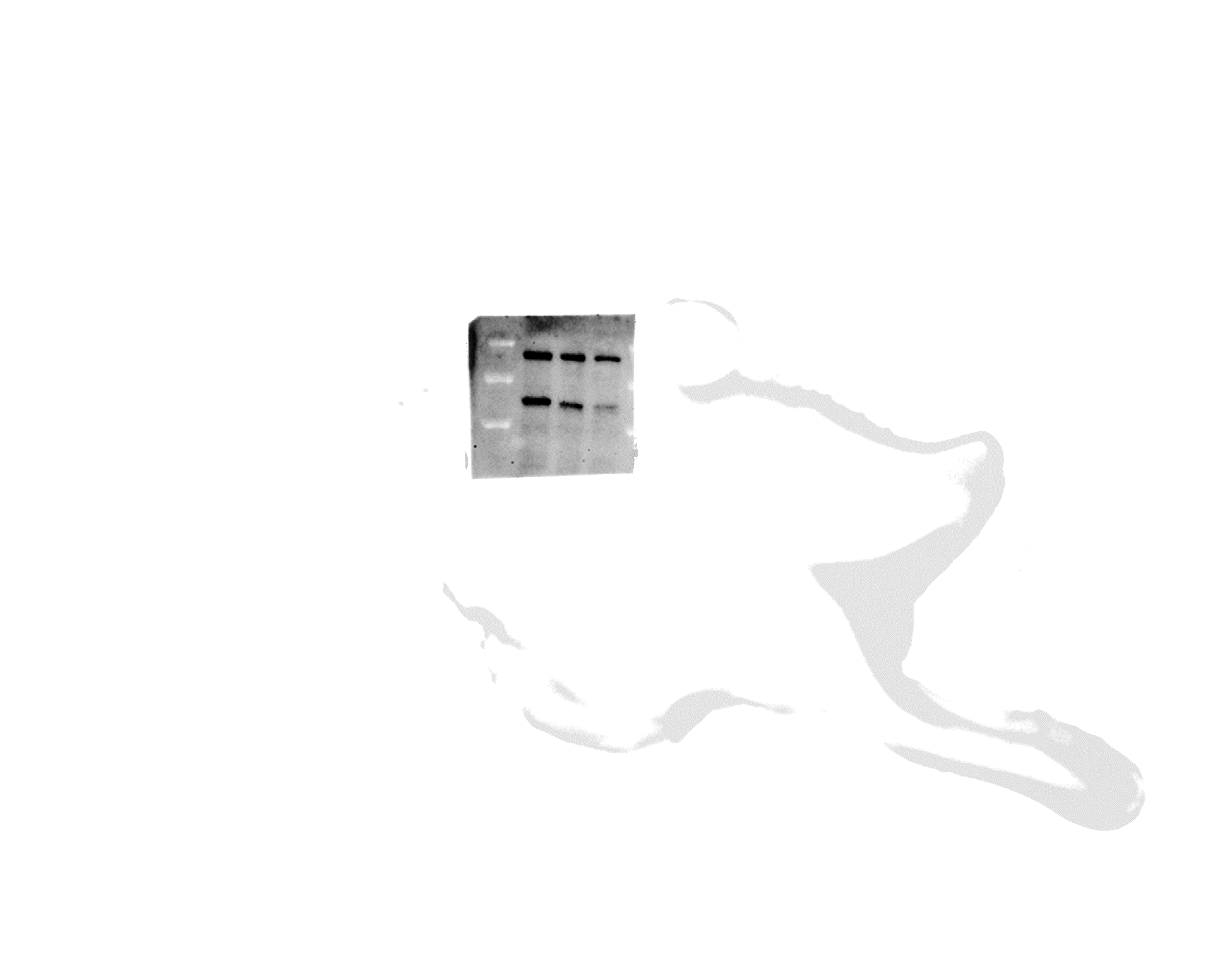

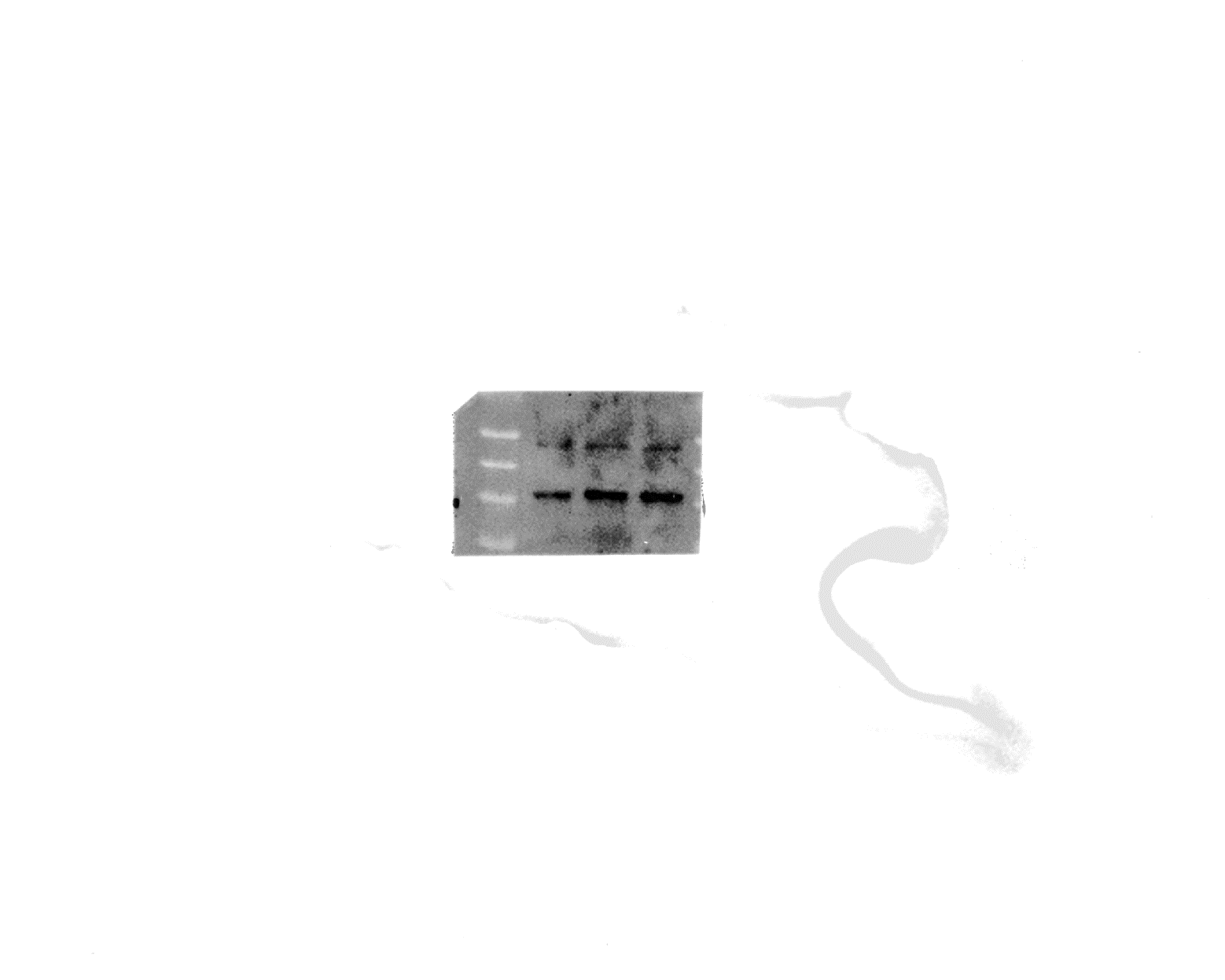


129KDa

37KDa

THBS1

Cyclin D1

METTL7A

METTL7A

siMETTL7A-2

siMETTL7A-2

siMETTL7A-1

siMETTL7A-1

Control

Control

Control

Control

Our membranes were cut immediately after transfer to incubate in different primary antibodies, which were probed individually for the proteins of interest, as appropriate for their molecular weights.
